# Supplementary material for: Nuclear lamina integrity is required for proper spatial organization of chromatin in Drosophila
Source: Nat Commun. 2019 Mar 12;10:1176. doi: 10.1038/s41467-019-09185-y (PMC6414625; doi:10.1038/s41467-019-09185-y)
Supplement: Supplementary file 1 — Supplementary Information [file 41467_2019_9185_MOESM1_ESM.pdf]

Supplementary Information

Nuclear lamina integrity is required for proper spatial organization of chromatin in *Drosophila*

Ulianov et al.

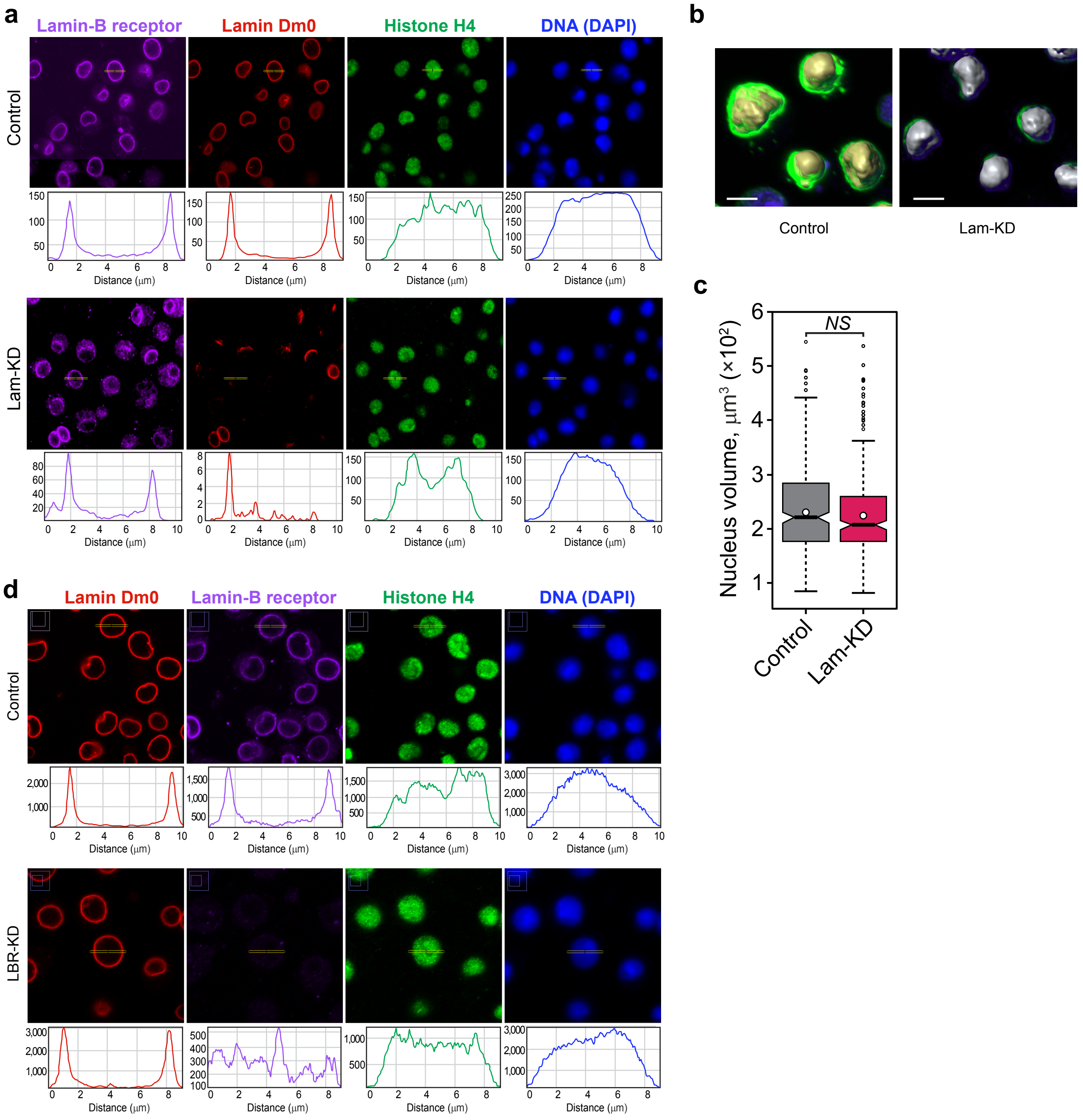

Supplementary Fig. 1 Immunostaining of chromatin in Lam-KD, LBR-KD and control cells. **a** A representative example of nuclei immunostained with antibodies against histone H4, LBR and lamin Dm0 in Lam-KD and control cells. Fluorescence intensity along the yellow-framed zone was measured using ImageJ software and presented below the images. **b** A representative example of nuclei immunostained with antibodies against lamin Dm0 (green) and counterstained by DAPI (blue) with the subsequent automatic reconstruction of the chromatin surface by DAPI staining using IMARIS software in Lam-KD or control S2 cells. Scale bar 5 µm. **c** Distribution of the volume of nuclei in Lam-KD (n=275) or control (n=275) S2 cells reconstructed by the LBR-stained NE using IMARIS software. *NS* – non-significant difference ($P > 0.05$ in a Wilcoxon test). Thick black lines and white dots represent median and average values, respectively. **d** A representative example of nuclei immunostained with antibodies against histone H4, LBR and lamin Dm0 in LBR-KD and control cells. Fluorescence intensity along the yellow-framed zone was measured using ImageJ software and presented below the images.

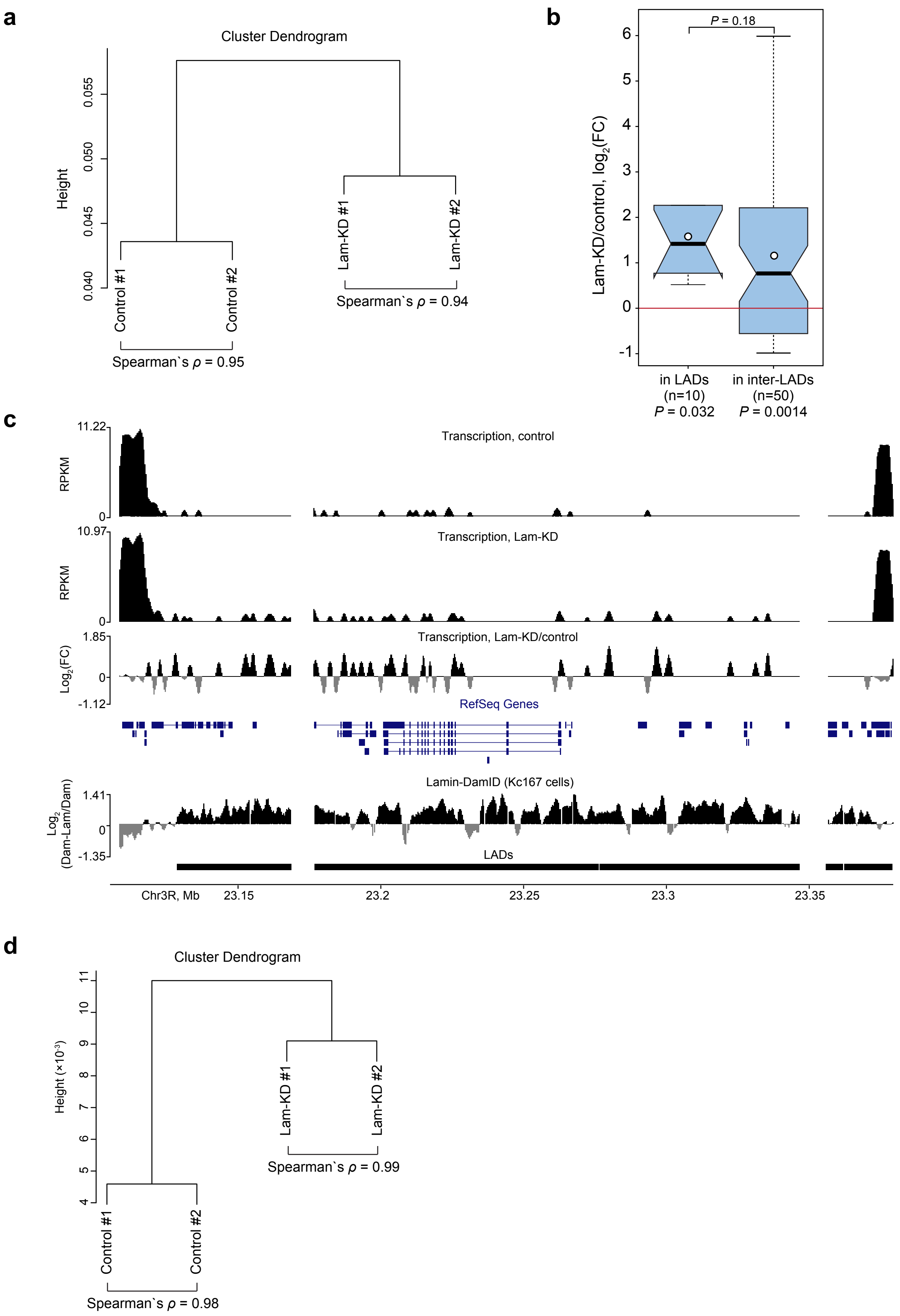

Supplementary Fig. 2 RNA-seq and ChIP-seq profiling in Lam-KD and control S2 cells. **a** Cluster analysis of biological replicates of the RNA-seq experiment. **b** Changes of gene expression for the differentially expressed genes in Lam-KD relative to control cells. The *P*-values for the comparison between "in LADs" and "in inter-LADs" groups, as well as for testing that average values in distributions exceed zero (the latter are shown below the box plots) were estimated in a Wilcoxon test. **c** A representative screenshot from the UCSC Genome Browser showing up-regulation of background transcription in LADs. **d** Cluster analysis of biological replicates of the ChIP-seq experiment.

a

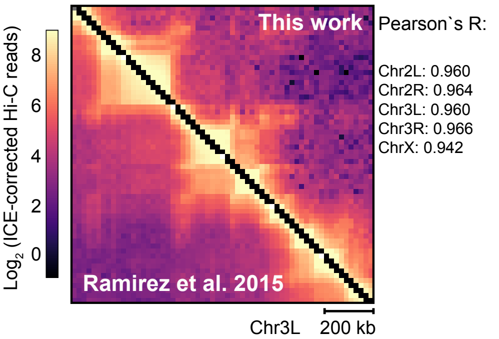

b

Chr		Control #1	Control #2	Lam-KD #1	Lam-KD #2
2L	Control #1		0.986	0.984	0.985
	Control #2	0.986		0.985	0.985
	Lam-KD #1	0.984	0.985		0.987
	Lam-KD #2	0.985	0.985	0.987	
2R	Control #1		0.986	0.983	0.984
	Control #2	0.986		0.984	0.984
	Lam-KD #1	0.983	0.984		0.987
	Lam-KD #2	0.984	0.984	0.987	
3L	Control #1		0.985	0.984	0.984
	Control #2	0.985		0.984	0.985
	Lam-KD #1	0.984	0.984		0.987
	Lam-KD #2	0.984	0.985	0.987	
3R	Control #1		0.987	0.984	0.984
	Control #2	0.987		0.984	0.985
	Lam-KD #1	0.984	0.984		0.987
	Lam-KD #2	0.984	0.985	0.987	
X	Control #1		0.966	0.965	0.964
	Control #2	0.966		0.964	0.965
	Lam-KD #1	0.965	0.964		0.967
	Lam-KD #2	0.964	0.965	0.967	

c

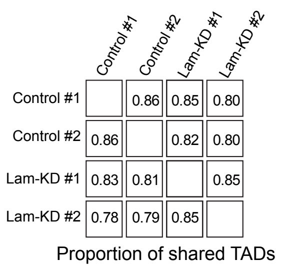

d **e** **f**

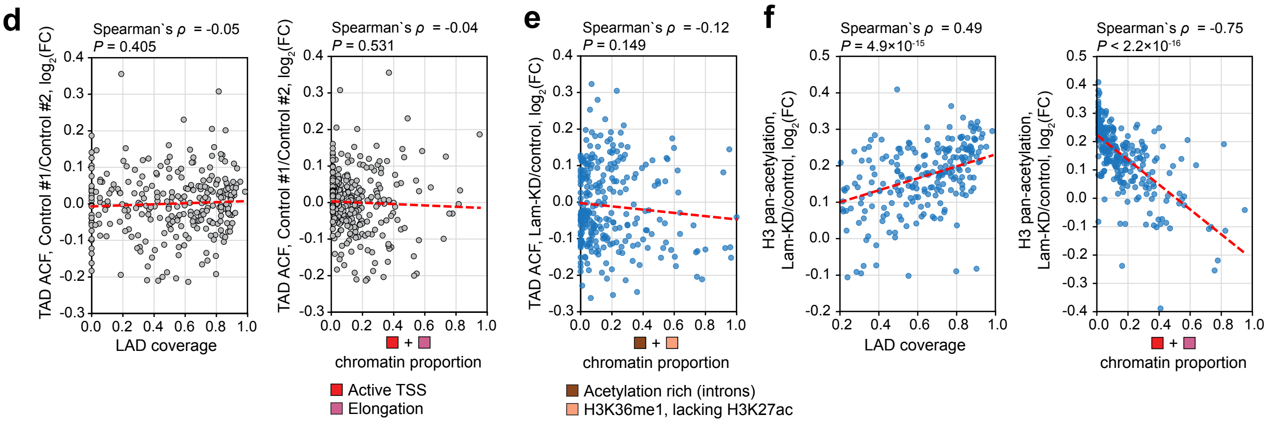

Supplementary Fig. 3 Hi-C analysis and characteristics of TADs from the four groups. **a** Comparison of heatmaps generated based on Hi-C data for control S2 cells in this work and for S2 cells in supplem. ref. 1. Pearson's correlation coefficients between ICE-corrected Hi-C matrices for each chromosome are shown to the right. **b** Pearson's correlation coefficients between ICE-corrected Hi-C matrices obtained in biological replicates of Hi-C experiments performed in this work. **c** Proportions of TADs located at the same position (±1 bin) in control and Lam-KD cells. **d** Intra-TAD ACF variability between replicates for control cells does not correlate with the LAD coverage (left panel) and the proportion of active chromatin (right panel) within TADs. **e** Intra-TAD ACF changes upon Lam-KD do not correlate with the proportion of "coral" plus "brown" chromatin types within TADs. **f** Changes of histone H3 pan-acetylation level in TADs upon Lam-KD positively correlate with the LAD coverage (left panel) and negatively correlate with the proportion of "red" plus "purple" chromatin types (supplem. ref. 2; right panel). Trend lines are in red.

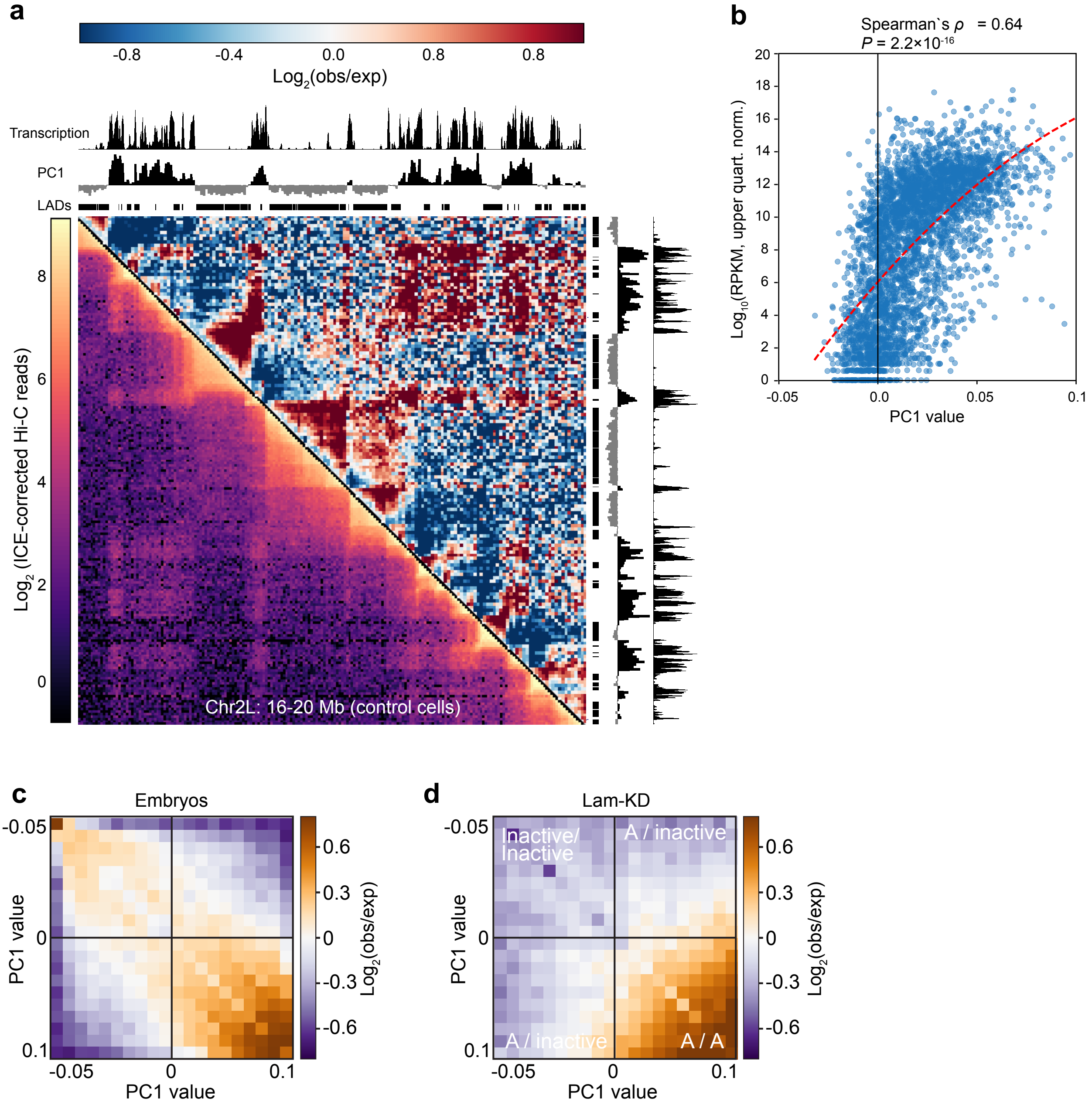

Supplementary Fig. 4 Identification of chromatin compartments. **a** ICE-corrected Hi-C map (left half) and observed/expected Hi-C matrix (right half) demonstrating the presence of A compartment manifested in the enhanced interactions between the transcriptionally active loci. **b** Transcription level within genomic bin correlates positively with the PC1 value. **c** Heatmap of intra-chromosomal contacts in embryos (from supplem. ref. 3) between genomic regions as a function of their PC1 values (saddle plot); heatmap shows $\log_2$ values of contact enrichment. **d** Saddle plot for Lam-KD S2 cells.

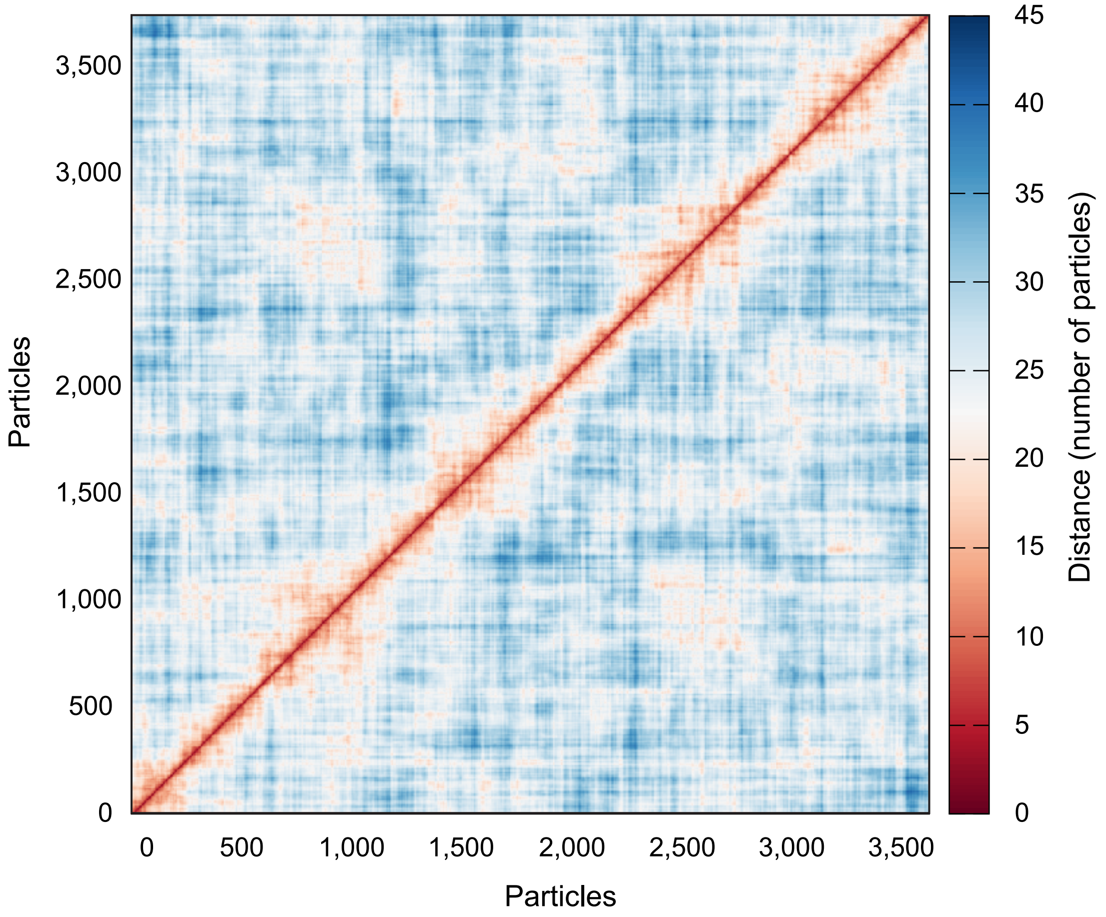

Supplementary Fig. 5 The presence of an interacting surface does not prevent the formation of TADs in a model polymer. Distance heat map of a model polymer obtained by the averaging of distance maps from 10 independent simulation runs.

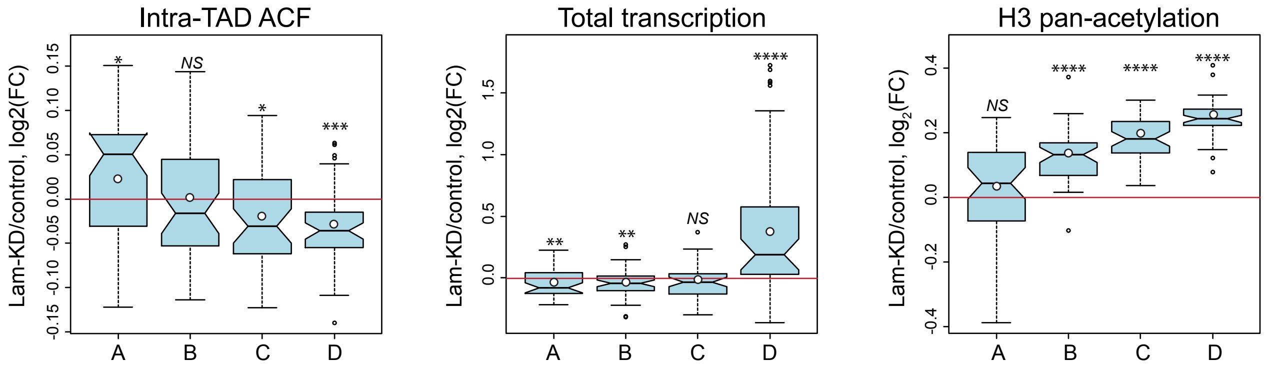

Supplementary Fig. 6 (related to Fig. 3) Strict criterion of TAD selection (i.e. all four ratios of replicates were changed in the same direction upon Lam-KD) does not affect the results of analysis. Changes of intra-TAD ACF (left panel), total transcription (middle panel) and H3 pan-acetylation (right panel) between Lam-KD and control cells in the four groups of TADs. The thick black lines and white dots represent median and average values, respectively. **** – P < 0.0001, *** – P < 0.001, ** – P < 0.01, * – P < 0.05, *NS* – non-significant difference (P > 0.05) in a Wilcoxon test.

Supplementary Table 1. Primers used in this study.

Gene or primer name	Direct primer (5'->3')	Reverse primer (5'->3')
**Primers for RT-PCR of genes from 60D LAD**		
SerT	GACATGACCACGCCCAGGTTCACG	TCCGATACTGATCTTCCGACGGGCA
CG3419	CATCGCACATATACAGCCTGCCCTTGC	TTGGTCCCGACTTCTTCACCTCGCA
CG42383	CCCGGAAACAGTTCATGGGACCAGG	GCGGAAAGGCGGTCATCAGGACAAA
prom	TGCTTCTCTGGGCTATTGGCACTCCG	GCTTGTCGCTGAACTGCTCGTCCGT
CG15873	CGGAACCCGTGGGAGAAAGCATGAA	CGCAGTCGGAAAGGGATTGGATGGT
CG15874	CAAAGGTTGCGGGAAATGGGCTTGT	GTCCAGGCTGCGGGCACTCCTCT
CG3483	AAGCAGTTCGCCCAGTTCGCCTACG	GCCACCGTGATCCTTTTGCGCTTGT
CG4563	TCAACCTGGGCGACCTGGGCTACTT	ATCTCGCATCCCGCACGCCTACC
CG13579	CTGCGAGCCCTTCTACAGCAAGCCA	GGTCGGATCGTTTAGCCGGAAGCG
CG3492	GCAGTGAAGCGCGGACCCTACACAA	TGTGGGATCGGATCTTGGGCATCG
CG3494	TGCCCAAAGATCTGCCCTTGCTCTCC	CAGCGTGGCAACTGGCGGAAGCGA
CG16837	CGTTCGTCAATCTCTTCGCCCATCG	CCAGGGTCATGTGGATTTCGCCCAT
CG13589	CACCCCTTGCTAAAATGACGAATGC	TTTCGCTGGTTCTATGAATGTGGCA
CG13590	CCTTACTGCCCCTTCTGTGCTTCGAGC	CCACCATCAACCACACCTGTCCCTATGAG
CG4589	CGGAACAATATGGGACACACGAGCAACA	CGACCCTGTCCACGACGATAACGGC
**Primers for RT-PCR of genes from other LADs**		
Eaat1	TACATTGGCATCATAAACTCATC	AACATCACAAGACCCAGGAC
CG30395	AGTTATACTTCAATGCACCTGTTT	ATGGGAGTCTTCGGGCTTAC
CG34391	GGCTAATGCTGCTTGAATGC	TGTGGGTAACCTGCTTGGAT
CG5162	ATCCTAACACCTACTGGCATAA	CAGGGTATCAACGAAACGAG
CG34370	AATCATCAGCCAATTCTAACTACC	TCTTCCTTAGCATCGCCCAC
Rim	AGCCGACACCATTACCACCT	CGAATGTTTGTCAGAATCCCT
wat	CGGCACCAGAGCTAATGTAT	CACCCTGAACACCCTTACGC
beat-Va	ATCCGTCACAAACAGAGCAT	TCTTTGGGGAAAACAACATC
Sls	CCACCATGATGTTGTTGCAC	CACTTCCGCTACCATCCATA
Byn	ACATTGGCGCTCACTATTTG	GAGGCACTGATCTTCACGAC
Goe	CTGTAGGACGACCAGAACCC	CATGATCCCACTAATTTGAGC
CG31814	CATTAGAGCATCTCGACCCA	GGGAATTGAAAAGGACTAAGTAAA
Fili	GGCAATGTGATGAGCGAACT	TGATTAAGGGCAGATATGAAAA
Mb1	TCAGTTATTGATAAATGGACGCA	AGTGGATAGCGGATGGAATG
**Primers for 2L 16,964,000-16,982,000 amplification (green FISH probe)**		
1	CCTCCATTTCCACCCACAGTTTCCCA	GCCCAAGTGCCACGAGCCTCAAATAA
2	TTATTTGAGGCTCGTGGCACTTGGGC	GCGATTTTCAGGACTCGGGGACTGG
3	CCAGTCCCCGAGTCCTGAAAATCGC	TTTTTGCTTTGACAACCCTGCCGCA
4	TGCGGCAGGGTTGTCAAAGCAAAAA	CCTTGTCCAGAGGATAAAAAACGGTGCCC
**Primers for 2L 17,310,000-17,328,000 amplification (red FISH probe)**		
1	GCCCACCACCCACTTTTTGGCTTTG	CCCTCTGACCCAACAGCACGTTTTTCA
2	GGGAGGGCGAACATTGTGGGATCAG	TTTGTCATTGTGGGTGCGTTGCTGC
3	GCAGCAACGCACCCACAATGACAAA	GAGAGCGAGCAAAAAGGCCGTGGAA
4	TTCCACGGCCTTTTTGCTCGCTCTC	GGAGCTCTTGTGAGGCCCGAACCAA
**Primers for lamin Dm0 dsRNA preparation**		
Direct primer	GAATTAATACGACTCACTATAGGGAGAATGTCGAGCAAATCCCGACGT	
Reverse primer	GAATTAATACGACTCACTATAGGGAGAGCGACTGCTTCAACTTGGCATC	
**Primers for LBR dsRNA preparation**		
Direct primer	GAATTAATACGACTCACTATAGGGAGACCCAGTCCAAGCAGCCCAGCC	
Reverse primer	GAATTAATACGACTCACTATAGGGAGAGGCAAAGGCACCCACCACTCGT	

Supplementary References

1. Ramírez, F. *et al*. High-affinity sites form an interaction network to facilitate spreading of the MSL complex across the X chromosome in Drosophila. *Mol*. *Cell* **60,** 146–162 (2015).
2. Kharchenko, P. V. *et al*. Comprehensive analysis of the chromatin landscape in Drosophila melanogaster. *Nature* **471,** 480–485 (2011).
3. Sexton, T. *et al*. Three-dimensional folding and functional organization principles of the Drosophila genome. *Cell* **148,** 458–472 (2012).
