# Supplementary material for: Nuclear lamina integrity is required for proper spatial organization of chromatin in Drosophila
Source: Nat Commun. 2019 Mar 12;10:1176. doi: 10.1038/s41467-019-09185-y (PMC6414625; doi:10.1038/s41467-019-09185-y)
Supplement: Supplementary file 3 — Reporting Summary [file 41467_2019_9185_MOESM3_ESM.pdf]

nature research

Corresponding author(s): Yuri Shevelyov, Sergey Ulianov

Last updated by author(s): Feb 11, 2019

Reporting Summary

Nature Research wishes to improve the reproducibility of the work that we publish. This form provides structure for consistency and transparency in reporting. For further information on Nature Research policies, see Authors & Referees and the Editorial Policy Checklist.

Statistics

For all statistical analyses, confirm that the following items are present in the figure legend, table legend, main text, or Methods section.

n/a	Confirmed	
☐	☒	The exact sample size (*n*) for each experimental group/condition, given as a discrete number and unit of measurement
☐	☒	A statement on whether measurements were taken from distinct samples or whether the same sample was measured repeatedly
☐	☒	The statistical test(s) used AND whether they are one- or two-sided<br>*Only common tests should be described solely by name; describe more complex techniques in the Methods section.*
☒	☐	A description of all covariates tested
☒	☐	A description of any assumptions or corrections, such as tests of normality and adjustment for multiple comparisons
☒	☐	A full description of the statistical parameters including central tendency (e.g. means) or other basic estimates (e.g. regression coefficient) AND variation (e.g. standard deviation) or associated estimates of uncertainty (e.g. confidence intervals)
☒	☐	For null hypothesis testing, the test statistic (e.g. *F*, *t*, *r*) with confidence intervals, effect sizes, degrees of freedom and *P* value noted<br>*Give P values as exact values whenever suitable.*
☒	☐	For Bayesian analysis, information on the choice of priors and Markov chain Monte Carlo settings
☒	☐	For hierarchical and complex designs, identification of the appropriate level for tests and full reporting of outcomes
☐	☒	Estimates of effect sizes (e.g. Cohen's *d*, Pearson's *r*), indicating how they were calculated

Our web collection on statistics for biologists contains articles on many of the points above.

Software and code

Policy information about availability of computer code

Data collection	Computer code for polymer modeling is available at GitHub (https://github.com/KPavell/dpd).
Data analysis	Computer code for polymer modeling is available at GitHub (https://github.com/KPavell/dpd).

For manuscripts utilizing custom algorithms or software that are central to the research but not yet described in published literature, software must be made available to editors/reviewers. We strongly encourage code deposition in a community repository (e.g. GitHub). See the Nature Research guidelines for submitting code & software for further information.

Data

Policy information about availability of data

All manuscripts must include a data availability statement. This statement should provide the following information, where applicable:

- Accession codes, unique identifiers, or web links for publicly available datasets
- A list of figures that have associated raw data
- A description of any restrictions on data availability

Raw and processed Hi-C, RNA-seq and ChIP-seq data were deposited in the GEO NCBI under the accession number GSE110082. DPD code is available at GitHub [https://github.com/KPavell/dpd]. The source data underlying Figs 1a, b, d–f, h, i, 2d, 3, 5b, c and Supplementary Fig. 1c are provided as a Source Data file. All other data supporting the findings of this study are available from the corresponding authors upon request.

Field-specific reporting

Please select the one below that is the best fit for your research. If you are not sure, read the appropriate sections before making your selection.

☒ Life sciences ☐ Behavioural & social sciences ☐ Ecological, evolutionary & environmental sciences

For a reference copy of the document with all sections, see nature.com/documents/nr-reporting-summary-flat.pdf

Life sciences study design

All studies must disclose on these points even when the disclosure is negative.

Sample size	No sample-size calculation was performed.
Data exclusions	No data were excluded from the analysis.
Replication	All experiments were performed at least in two biological replicates.
Randomization	Not applicable to this study.
Blinding	Analysis of FISH and immunostaining data was done with the blind experimental setup.

Reporting for specific materials, systems and methods

We require information from authors about some types of materials, experimental systems and methods used in many studies. Here, indicate whether each material, system or method listed is relevant to your study. If you are not sure if a list item applies to your research, read the appropriate section before selecting a response.

Materials & experimental systems

n/a	Involved in the study	
☐	☒	Antibodies
☐	☒	Eukaryotic cell lines
☒	☐	Palaeontology
☒	☐	Animals and other organisms
☒	☐	Human research participants
☒	☐	Clinical data

Methods

n/a	Involved in the study	
☐	☒	ChIP-seq
☒	☐	Flow cytometry
☒	☐	MRI-based neuroimaging

Antibodies

Antibodies used	Rabbit polyclonal anti-H3-pan acetylated antibodies, Active Motif, #39139;<br>Murine monoclonal anti-histone H4 antibodies, Abcam, ab31830;<br>Rabbit polyclonal anti-lamin Dm0 antibodies were provided by Paul Fisher (Stony Brook University School of Medicine);<br>Rabbit polyclonal anti-lamin C antibodies were provided by Paul Fisher (Stony Brook University School of Medicine);<br>Guinea-pig polyclonal anti-LBR antibodies were provided by Georg Krohne (Julius-Maximilians-Universität Würzburg);<br>Murine monoclonal anti-beta Actin antibodies, Abcam, ab8224.
Validation	Broad reactivity of anti-H3-pan acetylated antibodies is confirmed by the manufacturer<br>https://www.activemotif.com/catalog/details/39139/histone-h3ac-pan-acetyl-antibody-pab-1

Eukaryotic cell lines

Policy information about cell lines

Cell line source(s)	S2 cell line was obtained from the IMG RAS collection;<br>Kc167 cell line was obtained from the Drosophila Genomics Resource Center;<br>OSC line was kindly provided by Mikiko Siomi (The University of Tokyo).
Authentication	None of the cell lines used were authenticated.
Mycoplasma contamination	Cell lines were not tested for mycoplasma contamination.
Commonly misidentified lines<br>(See ICLAC register)	Commonly misidentified cell lines were not used in this study.

ChIP-seq

Data deposition

☒ Confirm that both raw and final processed data have been deposited in a public database such as GEO.

☒ Confirm that you have deposited or provided access to graph files (e.g. BED files) for the called peaks.

Data access links<br>*May remain private before publication.*	GEO NCBI accession number GSE110082.
Files in database submission	GSM2977368 S2 control_Hi-C_rep1<br>GSM2977369 S2 control_Hi-C_rep2<br>GSM2977370 S2 lamin Dm0 KD_Hi-C_rep1<br>GSM2977371 S2 lamin Dm0 KD_Hi-C_rep2<br>GSM3449340 Chip_LacZ_rep1<br>GSM3449341 Chip_LacZ_rep2<br>GSM3449342 Chip_Lam_rep1<br>GSM3449343 Chip_Lam_rep2<br>GSM3449344 Input_LacZ_rep1<br>GSM3449345 Input_LacZ_rep2<br>GSM3449346 Input_Lam_rep1<br>GSM3449347 Input_Lam_rep2<br>GSM3449348 RNA_LacZ_rep1<br>GSM3449349 RNA_LacZ_rep2<br>GSM3449350 RNA_Lam_rep1<br>GSM3449351 RNA_Lam_rep2
Genome browser session<br>(e.g. UCSC)	Not applicable.

Methodology

Replicates	Hi-C, RNA-seq, ChIP-seq were performed in two biological replicates.
Sequencing depth	Hi-C libraries were sequenced on the Illumina HiSeq 2000 resulting in 30-40 million 75-bp paired-end reads.<br>RNA-seq libraries were sequenced on the Illumina HiSeq 2000 resulting in 8.4-9.4 million 75-bp single-end reads.<br>ChIP libraries were sequenced on the Illumina HiSeq 2000 resulting in 3.1-3.4 million 75-bp single-end reads.
Antibodies	Rabbit polyclonal anti-H3-pan acetylated antibodies, Active Motif, #39139
Peak calling parameters	Not applicable. We did not use peak calling procedure. Instead, we analyzed ratios of ChIP-seq profiles between Lam-KD and control S2 cells. We have deposited these ChIP-seq profiles in GEO NCBI under accession number GSE110082 as BEDgraph format files.
Data quality	Not applicable.
Software	ChIP-seq reads were mapped to the D. melanogaster reference genome (version dm3) using Bowtie 2 v2.2.1 (with the --very-sensitive option). Reads with low mapping quality were removed using SAMtools with option -q 30. Duplicate reads were removed using SAMtools rmdup. We calculated ChIP and input signals in 1-kb genomic bins using BEDtools v2.16.2.
